# Projected climate and agronomic implications for corn production in the Northeastern United States

**DOI:** 10.1371/journal.pone.0198623

**Published:** 2018-06-11

**Authors:** Rishi Prasad, Stephan Kpoti Gunn, Clarence Alan Rotz, Heather Karsten, Greg Roth, Anthony Buda, Anne M. K. Stoner

**Affiliations:** 1 Pasture Systems and Watershed Management Research Unit, USDA/Agricultural Research Service, University Park, Pennsylvania, United States of America; 2 Crop, Soil and Environmental Sciences Department, Auburn University, Auburn, Alabama, United States of America; 3 Plant Science Department, The Pennsylvania State University, University Park, Pennsylvania, United States of America; 4 Climate Science Center, Texas Tech University, Lubbock, Texas, United States of America; Fred Hutchinson Cancer Research Center, UNITED STATES

## Abstract

Corn has been a pillar of American agriculture for decades and continues to receive much attention from the scientific community for its potential to meet the food, feed and fuel needs of a growing human population in a changing climate. By midcentury, global temperature increase is expected to exceed 2°C where local effects on heat, cold and precipitation extremes will vary. The Northeast United States is a major dairy producer, corn consumer, and is cited as the fastest warming region in the contiguous U.S. It is important to understand how key agronomic climate variables affect corn growth and development so that adaptation strategies can be tailored to local climate changes. We analyzed potential local effects of climate change on corn growth and development at three major dairy locations in the Northeast (Syracuse, New York; State College, Pennsylvania and Landisville, Pennsylvania) using downscaled projected climate data (2000–2100) from nine Global Climate Models under two emission pathways (Representative Concentration Pathways (RCP) 4.5 and 8.5). Our analysis indicates that corn near the end of the 21^st^ century will experience fewer spring and fall freezes, faster rate of growing degree day accumulation with a reduction in time required to reach maturity, greater frequencies of daily high temperature ≥35°C during key growth stages such as silking-anthesis and greater water deficit during reproductive (R1-R6) stages. These agronomic anomalies differ between the three locations, illustrating varying impacts of climate change in the more northern regions vs. the southern regions of the Northeast. Management strategies such as shifting the planting dates based on last spring freeze and irrigation during the greatest water deficit stages (R1-R6) will partially offset the projected increase in heat and drought stress. Future research should focus on understanding the effects of global warming at local levels and determining adaptation strategies that meet local needs.

## Introduction

Corn is an important crop in American agriculture, offering many possibilities for feeding and fueling a growing world population. However, future corn production will potentially face vagaries of extreme weather in a warming climate led by anthropogenic greenhouse gas emissions [1–2]. These emissions are currently the highest in human history and are expected to continue in coming decades [3]. While mean annual temperatures have already increased throughout the world, the global temperature is expected to further increase by 2°C by 2050, and their local effects on heat, cold and precipitation extremes will vary widely with regional differences in geography and landscape features [4].

Corn production in the Northeast United States (NE US) is deemed to suffer from the impacts of climate change like other corn growing regions of the US; however, the NE region draws special attention for two reasons. First, the NE is a major dairy region and corn provides a major feed for the dairy industry [5–6]. In 2017, New York and Pennsylvania ranked third and seventh in total milk production across the United states producing an average 5490 and 4845 million kg of milk, respectively [5]. Second, the NE US is cited to be the fastest warming region in the contiguous US [7–9]. Average ambient temperature in the NE is projected to warm by 3°C when the global average temperature reaches 2°C by 2050 [4]. Most climate change studies on corn have been carried out in the Midwest and Great Plains with little attention to NE US; hence, there is a need to evaluate the local risks of extreme climate on corn production in this region [10–12]. This must include the evaluation of adaptation strategies suited for the local conditions to enhance regional and national food security. While the majority of climate change studies on corn have used either simulation or statistical models to evaluate yield losses, none of them have focused on aspects of corn growth and development from an agronomic viewpoint as influenced by extreme climate conditions [12–14]. Extreme climate adversely affects corn growth and development and ultimately reduces yield [15–16]. Extreme climate such as increased occurrences of warm temperature extremes, reduced cold extremes, and increased number of heavy precipitation events have been observed since the post-industrial era [3, 17–18] and are expected to increase as climate change continues to accelerate [3].

Corn responses to extreme temperature (defined as daily temperatures below the minimum, Tmin ≤0°C, and above the optimum, Tmax ≥35°C) vary among vegetative and reproductive stages. For example, rising air temperature during the pollination stage causes ambient saturation vapor pressure to increase exponentially [19] resulting in a high vapor pressure deficit and very dry condition. As a result, the pollen dries prior to silk reception making them sterile [20]. Corn pollen loses its viability with exposure to temperatures above 35°C [21–24]. Additionally, a temperature increase from 30 to 35°C during the endosperm division phase reduces the potential kernel growth rate along with the final kernel size, significantly reducing yield [25]. Other effects of extreme temperatures (both cold and hot) include frost kill with crop failure [26], damage to tissue and enzymes [27], lower net photosynthesis rates [28] and faster rates of crop development due to greater accumulation of heat energy, which leads to yield losses [29].

Precipitation is another important climate variable that affects yield in non-irrigated agriculture. The associated impact of precipitation is growth-stage-specific and more apparent when elevated temperatures increase evapotranspiration rates and extended agricultural drought periods increase water deficit. Drought conditions resulting from water deficit can cause reduced corn growth by allocating more carbon to the root system, reducing leaf expansion and photosynthesis, and accelerating senescence [30]. Several studies have demonstrated the importance of growth-stage-specific precipitation effects on corn yield [31–32]. Additionally, changes in temperature and precipitation patterns also affect the timeliness of field operations, planting dates, and suitability for harvesting the crop at a desired grain moisture level [33].

Although several global and regional studies have evaluated heat and water stress effects on corn yield using historical weather data [11, 34–35], there is less information on growth-stage-specific anomalies in corn production at local levels. Further, warming trends in the US are not spatially and temporally uniform [e.g. 35]. Therefore, local evaluations of climate change effects on corn production are important. Examining temperature anomalies, water deficit periods, and frost occurrences (early and late) during the corn growing season are of great importance as these factors are strongly associated with yield. Indeed, studies have indicated the need to conduct area-specific assessments of future corn production and its sensitivities to different projections of climate change [11, 15, 36].

**Fig 1:**
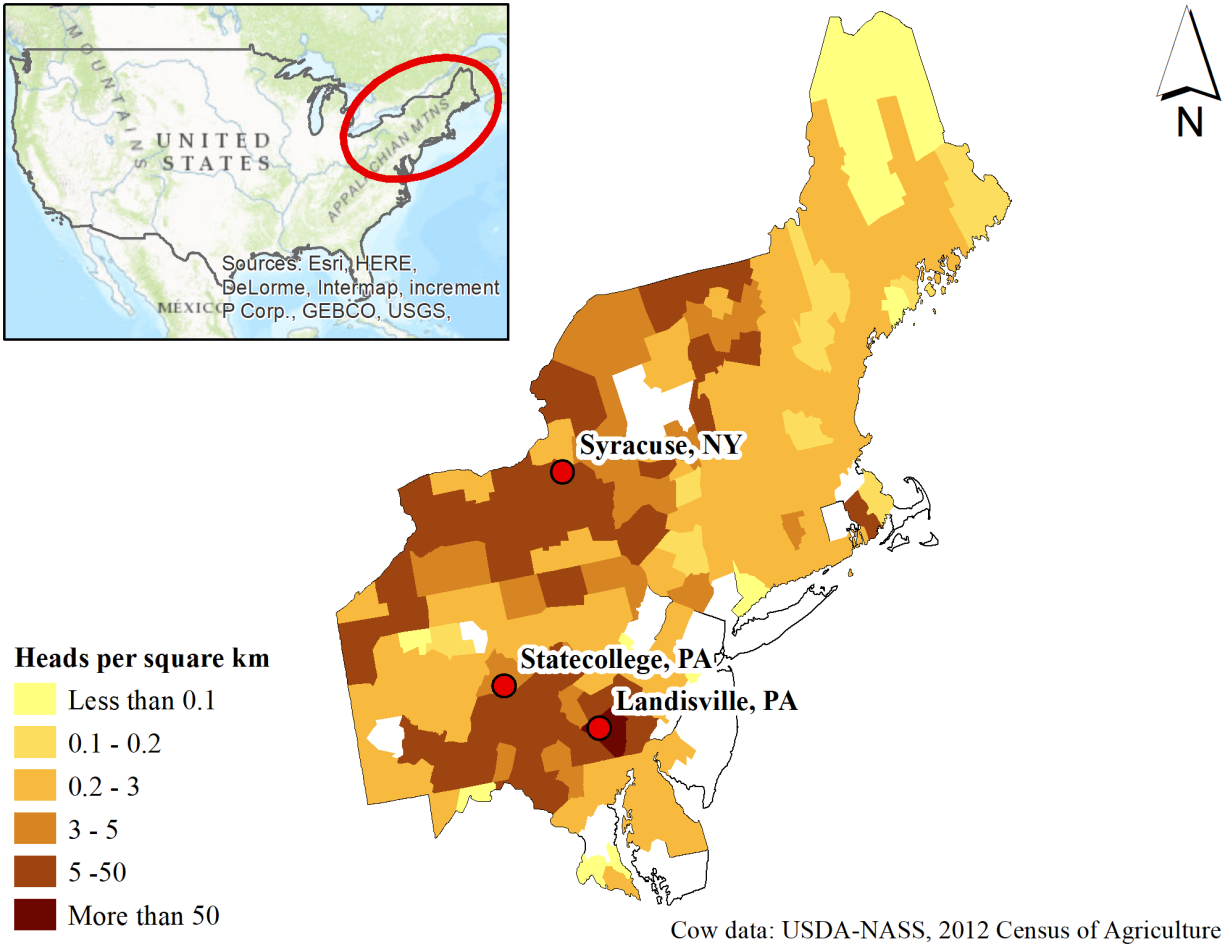
Corn study sites located in major dairy regions of the Northeast United States.

In this research, we examine the potential local impacts of changing climate on corn production from an agronomic viewpoint, at three spatially distinct locations in representative corn and dairy regions in the NE US (Syracuse, New York; State College, Pennsylvania and Landisville, Pennsylvania) (Fig 1) under two greenhouse gas Representative Concentration Pathways (RCP) 4.5 (reduced or low emission scenario) and RCP 8.5 (business-as-usual or a high emission scenario where greenhouse gas concentrations reach a level three times greater than current values at the end of 21st century (2080–2100)). Specifically, we explored five agronomically important considerations linked to corn production and yield: (1) trends for last spring freeze dates and their effect on future corn-planting dates, (2) Growing degree days (GDD, is a heat index used for describing the biological stage progression of a crop until it reaches maturity [37]) accumulation rate for the three latitudinal coordinates in the NE US, (3) effect of changing GDD rate on time to reach corn maturity, (4) days during the vegetative and reproductive phases of corn with maximum temperature exceeding the critical temperature of 35°C or less than the minimum temperature of 0°C, and (5) the deficit between potential evapotranspiration (PET) and precipitation at different corn growth stages.

## Methods

### Data acquisition and downscaling of climate projections

Projected daily climate data (maximum and minimum temperatures, precipitation and solar radiation) corresponding to RCP 4.5 and RCP 8.5 for years 2000 to 2100 were obtained from 9 Global Climate Models (GCMs). The nine GCMs are part of the fifth phase of the Coupled Model Intercomparison Project (CMIP5) (Table 1). The projected climate data were statistically downscaled to station-level resolution using the Asynchronous Regional Regression Model (AARM) [38]. The stations selected for this study included Landisville, Pennsylvania (40.10 latitude and -76.41 longitude), State College, Pennsylvania (40.80 and -77.87), and Syracuse, New York (43.1 and -76.1). Briefly, the ARRM uses piecewise regression to quantify the relationship between observed and modelled historical quantiles of temperature and precipitation to downscale projected future daily climate. Stoner et al. [38] evaluated the use of ARRM in downscaling daily minimum and maximum temperature and precipitation data for 20 stations representing diverse climate zones in North America and found the method to be efficient in simulating extremes and highly generalizable across multiple variables, regions, and climate model inputs.

**Table 1:** Coupled Model Intercomparison Project Phase 5 (CMIP5) climate models used in the present study.

Model acronym	Host institution
CCSM4	National Center for Atmospheric Research, (NCAR), USA
CNRM-CM5	Centre National de Recherches Meteorologiques, Meteo-France
CSIRO-Mk3-6-0	Australian Commonwealth Scientific and Industrial Research Organization
HadGEM2-CC	UK Met Office Hadley Centre
INMCM4	Institute for Numerical Mathematics, Russia
IPSL-CM5A-LR	Institut Pierre-Simon Laplace, France
MPI-ESM-LR	Max Planck Institute for Meteorology, Germany
MRI-CGCM3	Meteorological Research Institute, Japan
MIROC5	Model for Interdisciplinary Research on Climate, Japan

### Growing degree day (GDD) calculation

Projections of maximum and minimum daily temperatures were used to calculate the heat energy accumulation (or growing degree days) for corn. The GDD was calculated using Eq (1).

$$GDD=\frac{Tmax+Tmin}{2}-Tbase$$ (1)

where Tmax is the maximum daily air temperature limited to a maximum value of 30°C and Tmin is the minimum daily air temperature set to a minimum value of 10°C. The base temperature (Tbase) used for corn is 10°C [39].

### Corn growth stage estimation

Corn growth stages were defined based on Abendroth et al [40] using the U2U corn growing degree day tool [39]. The U2U corn GDD tool was built upon the relationship from field studies documented in Abendroth et al., [40]; Neild and Newman, [41] and data observations from 557 Pioneer, 56 Golden harvest and 69 Northrup corn hybrid varieties [39]. The tool has been successfully used in major corn producing states as well in research [39, 42]. The major corn growth stages predicted were VE (emergence), V6 (six leaf stage), V10 (10 leaf stage), R1 (silking) and R6 (black layer or physiological maturity). Briefly, the tool uses cultivar relative maturity (CRM, an index used by commercial seed companies to compare between hybrids and is based on GDD accumulations from planting to kernel black layer [43]; and GDD to predict corn growth stage. Crop emergence is assumed to occur upon accumulation of 105 GDD and leaf appearance from VE to Vn is estimated to occur every 84 GDD. Silking and black layer occurrences are predicted according to the following linear relationships:

SilkingGDD=192.8+10.66CRM (2)

BlackLayer=129.1+22.8CRM (3)

Growing degree days, CRM, and planting dates for the corn cultivars used in this study are listed in Table 2. The cultivars were classified as full and short season corn based on the GDD requirement to reach physiological maturity. The selected cultivars and planting dates are typical of the NE region [44–45]. Since corn is very sensitive to extreme heat and drought in the reproductive period, we explored the occurrences of extreme weather events by dividing the period between R1 and R6 stages into 160 GDD intervals.

**Table 2:** Characteristics of common corn cultivars planted in the Northeast United States.

Location	Corn cultivar (GDD)	Maturity rating	Planting date
Landisville, PA	Full season (2700)	114	25-Apr
	Short season (2350)	105	10-May
State College, PA	Full season (2500)	108	1-May
	Short season (2350)	100	15-May
Syracuse, NY	Full season(2350)	105	5-May
	Short season (2000)	85	20-May

### Extreme temperature frequency calculation

Corn pollen viability is greatly reduced at temperatures above 35°C, and the crop suffers from freeze injury or frost kill at temperatures below 0°C [19, 24]. Using the projected climate data, we calculated the potential rates of corn exposure (number of days) to temperatures beyond the two critical points (35°C and 0°C) during each growth stage. To achieve this task, we analyzed the daily Tmax and Tmin data predicted by each climate model for the two RCP and three locations, and calculated the median frequency counts of Tmax ≥ 35°C and Tmin ≤ 0 °C for each growth stage for the full and short season corn.

### Water deficit calculation

Water deficit (WD) is an indicator of potential water stress severity, which was estimated by taking the difference between PET and precipitation. To estimate the deficit between PET and precipitation for each growth stage, we first calculated the PET using the Priestly-Taylor method [46]. This method requires mean daily temperature (°C) and daily radiation (W m^-2^) as inputs. The mean daily temperature was calculated by taking the average of daily maximum and minimum temperatures. Daily radiation values were obtained from the 9 GCMs. Daily PET and precipitation data were cumulated for each growth stage and the difference was determined to obtain the water deficit by location, RCP and climate model. Although daily deficits can affect maize physiology but for practical purpose, the cumulative deficit has stronger influences on yield and yield parameters [47–48]. Hence, the daily PET and precipitation data were cumulated for each growth stage to determine water deficit.

### Identification of last spring freeze

Freezing temperatures can result in poor emergence due to injury caused by seeds imbibing cold water. We analyzed the projected daily Tmin data to determine the date of last spring freeze (Tmin ≤ 0°C) predicted for each year by each climate model for each location and RCP. We divided the Tmin data of each year in two halves (January to June and July to December) and used the data from January to June to identify the last freeze date. To identify the last spring freeze date we developed an algorithm in R software that searched the date of last occurrence of below zero degree temperature (i.e. Tmin ≤ 0 °C) between Januarys to June.

### Planting date sensitivity to spring freeze

We also examined how planting dates in future be potentially affected by the last date of spring freeze under the two RCPs at the three locations for the period 2000 to 2100. The current or business-as-usual planting dates (BAUPD) are one to two weeks after the last spring freeze dates (Table 2). To understand if BAUPD will be appropriate in the future, we carried a sensitivity test around planting dates by selecting two additional planting date scenarios. In the first scenario, we considered shifting the planting date 14 days prior to the occurrence of last spring freeze. This scenario is also a recommendation documented in the Penn State Agronomy Guide [45] according to which corn could be planted 10 to 14 days prior to the average date of last spring freeze. In the second scenario, we considered the planting date to be one day after the last spring freeze. The rationale behind the second scenario was to examine if planting corn immediately after the last spring freeze was more favorable than the BAUPD.

We also wanted to understand the influence of planting dates on corn maturity length, frequencies of exposure to high (Tmax ≥ 35°C) and low temperature (Tmin ≤ 0°C) and water deficits in corn growth stages. Using the three planting dates (BAUPD, planting 14 days prior to the occurrence of last spring freeze (scenario1), and planting 1 day after the last spring freeze (scenario2)), we examined (1) the trend of changes in corn maturity length for short and full season cultivars, (2) trend in the number of days with Tmax ≥ 35°C and Tmin ≤ 0°C in corn growth stages for the two corn cultivars, and (3) trend in water deficit in growth stages for both corn cultivars.

### Data organization and statistical analysis

The climate data from the nine GCMs representing two emission pathways from years 2000 to 2100 were aggregated by location. A long-term trend analysis was done for the corn maturity length, frequency of Tmax ≥ 35°C and Tmin ≤ 0°C, and the water deficit for each corn stage for the three planting dates (BAUPD, scenario1, scenario2). We evaluated the trends using the rank based non-parametric Mann-Kendall test [49–50]. The rate of change (slope) for each time series was determined using the Theil-Sen slope method [51–52], which calculates the median slope of all possible pairs of points in the data set. Data were evaluated for serial correlation and, if present, were de-trended using the Zhang method [53]. All data analysis and graphing were completed using the R software environment (version 3.3.1, R Development Core Team, 2016) including the Mann-Kendall tests and Theil-Sen slope calculations (R zyp package; [54]). All trends were considered statistically significant at α = 0.05 and only the median values of the trends determined for the nine models are reported throughout the manuscript.

## Results

### Spring freeze and future corn planting dates

**Fig 2:**
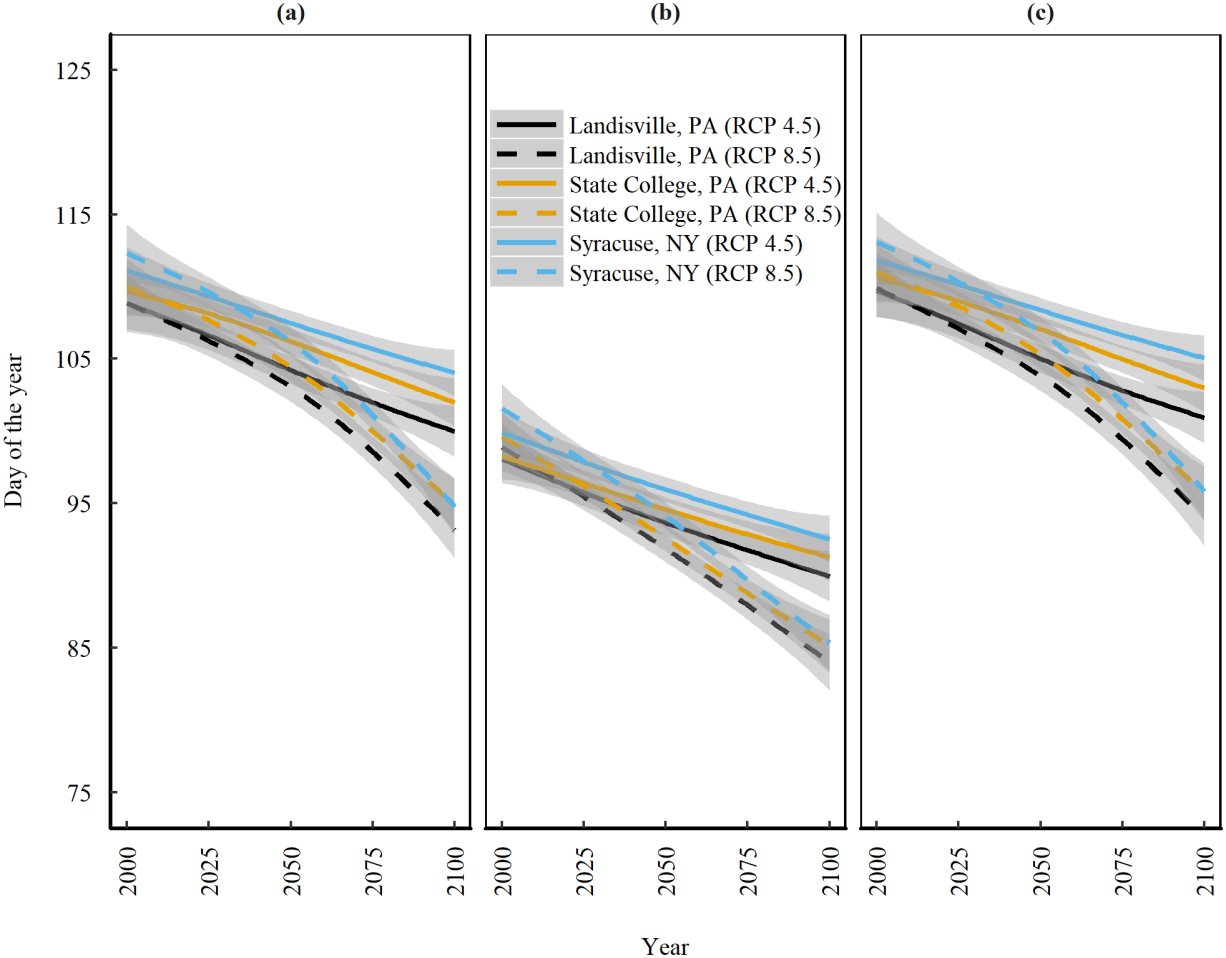
**Trends for projected changes in planting date in the 21**^**st**^ **century** as determined by (a) Last spring freeze. (b) 14 days before the last spring freeze. (c) One day after the last spring freeze, at three locations in the Northeast United States under emission scenarios of RCP 4.5 and 8.5. Solid and dashed lines are median trends of data predicted by nine Global Climate Models and shaded areas represent standard error of the mean.

The last spring freeze has historically been observed between April 16—April 30 in Landisville, May 1 –May 15 in State College, and May 21—May 31 in Syracuse [55–56]. The projections indicate that the last spring freeze will recede at all three locations under both RCP 8.5 and RCP 4.5 (Fig 2a) and the recession is stronger under RCP 8.5 than RCP 4.5 (2 day decade^-1^ vs. 1 day decade^-1^ (values are median of all models); Table 3). On a median scale (of all models) the last spring freeze under RCP 8.5 is expected to occur as early as the 94th day of the year (1st week of April) at Landisville and 95th day of the year at State College and Syracuse towards the end of the 21st century. Southern regions have been historically warmer than the northern and central parts of the NE [57]. The faster recession of spring freeze in northern and central part of NE (Syracuse and State College) indicate that these regions are warming rapidly such that they will be equally warmer as the southern regions of NE (Landisville). The rate of recession of spring freeze under RCP 8.5 at Syracuse, State College and Landisville are -1.7, -1.6 and -1.8 day decade-1, respectively. Lu et al [58] also reported a significant increasing trend at annual, seasonal, and monthly time scales from 1978 to 2012 for central Pennsylvania. They reported that annual mean temperatures increased at a steady rate of 0.38°C per decade.

**Table 3:** Projected median recession rate of last spring freeze (days per decade) at three locations in the Northeast United States under two emission scenarios during the 21^st^ century.

Location	RCP 4.5	RCP 8.5
Landisville	-1.2	-1.8
State College	-1.0	-1.6
Syracuse	-1.1	-1.7

Earlier timing of the last spring freeze has a significant impact on possible corn planting dates. The projections on last spring freeze suggest that farmers will have the opportunity to plant earlier at all three locations compared to BAUPD (Fig 2a). We assume that BAUPD stays the same over time. The change in median planting date according to scenario 1 and scenario 2 are presented in Fig 2b and 2c. Planting according to scenario 1 under RCP 8.5 is expected to occur sooner than under RCP 4.5 and could be as early as the 84th day of the year (beginning of the fourth week of March) at Landisville and the 85th and 86th day of the year (end of the fourth week of March) at State College and Syracuse, respectively, towards the end of 21st century (2080–2100). Planting dates according to scenario 2 under RCP 8.5 could be as early as the 93rd day of the year (beginning of the first week of April) at Landisville and the 96st day of the year (end of the first week of April) at State College and Syracuse towards the end of 21st century (2080–2100).

We also looked into trends in March precipitation, which could be an important climate factor driving soil moisture and machine trafficability affecting corn-planting under RCP 8.5. Our analysis revealed that at all three locations, while the March precipitation shows an increasing trend (2.1 mm decade^-1^), the PET also increases at a median rate of 2.5 mm decade^-1^. Greater PET than precipitation at all three locations confirm that a water deficit is projected to occur so trafficability of machinery across the soil surface might not be an issue for earlier planting.

### Time required to attain physiological maturity in corn

**Fig 3:**
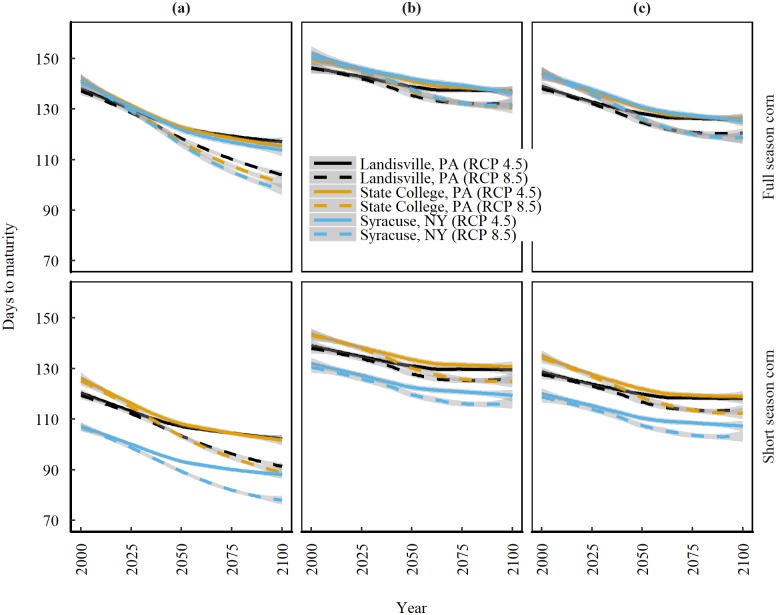
**Trends of projected days to reach physiological maturity in the 21st century for full and short season corn** based on (a) business-as-usual planting dates. (b) Planted 14-days before the last spring freeze. (c) Planted 1-day after the last spring freeze, at three locations in the Northeast United States under emission scenarios of RCP 4.5 and 8.5. Solid and dashed lines are median trends of data predicted by nine Global Climate Models and shaded areas represent standard error of the mean.

Corn (both full and short season) is expected to experience faster rate of GDD accumulation if planted using BAUPD under RCP 8.5 as compared to RCP 4.5 at all three locations in 21^st^ century (Fig 3a, Table 4). Consequently, corn maturity will shorten in the future (1.6 times faster under RCP 8.5 compared to RCP 4.5). The corn is also expected to experience a location difference for attaining the physiological maturity in the 21^st^ century (2050 and beyond). On an average, corn maturity is expected to decrease at a rate of 3 days per decade at Landisville, 3.9 at State college and 3.7 at Syracuse under RCP 8.5 in 21^st^ century. The faster decrease in corn maturity in central and northern locations of the NE (State College and Syracuse) compared to the most southerly location (Landisville) is a response to the faster warming rates in northern locations.

**Table 4:** Projected rates of reduction in time to corn maturity (days per decade) in the 21^st^ century for two corn cultivars planted according to business-as-usual date, scenario-1 (14 days before the last spring freeze) and scenario-2 (1 day after the last spring freeze) at three locations in the Northeast United States under two emission scenarios. Rates are reported as median values across projected data from 9 Global Climate Models.

		Landisville		State College		Syracuse	
Planting date	Corn cultivar	RCP 4.5	RCP 8.5	RCP 4.5	RCP 8.5	RCP 4.5	RCP 8.5
Business-as-usual	Full season	-2.0	-3.3	-2.5	-4.2	-2.8	-4.5
	Short Season	-1.7	-2.7	-2.2	-3.7	-1.8	-3.0
Average Business-as-usual		-1.8	-3.0	-2.4	-3.9	-2.3	-3.7
Scenario 1	Full season	-1.3	-1.7	-1.7	-2.6	-2.1	-2.7
	Short Season	-1.1	-1.4	-1.4	-2.2	-1.2	-1.5
Scenario 2	Full season	-1.2	-1.9	-2.0	-2.9	-2.2	-3.2
	Short Season	-1.0	-1.5	-1.4	-2.3	-1.3	-1.6
Average Scenario 1 & 2		-1.1	-1.6	-1.6	-2.5	-1.7	-2.2

Adjusting the planting dates shows a potential to reduce the time required for attaining maturity (Fig 3b and 3c; Table 4). Planting corn based on the last spring freeze date (Scenario 1 or Scenario 2) will potentially slow the faster GDD accumulation rate which in turn will slow the corn maturity rate by an average of 1.7 fold compared to BAUPD under RCP 8.5. If planted according to scenario 1 or scenario 2, the average median days for corn maturity would potentially decrease at a rate of 1.6 days per decade at Landisville, 2.5 days per decade at State College and 2.2 days per decade at Syracuse under RCP 8.5.

We also found a difference in maturity shrinkage between full and short season corn (Table 4). Full season corn will potentially mature 1.3 times faster than short season corn when planted according to BAUPD under RCP 8.5 (Fig 3, Table 4). Adjusting the planting dates according to scenario 1 will reduce the time to maturity of full season corn by 1.7 times and short season corn by 1.5 times compared to BAUPD under RCP 8.5. Similarly, adjusting the planting dates according to scenario 2 will potentially reduce the time to maturity of full season corn by 1.3 times and short season corn by 1.7 times compared to BAUPD under RCP 8.5.

### Extreme temperature frequencies at corn growth stages

**Fig 4:**
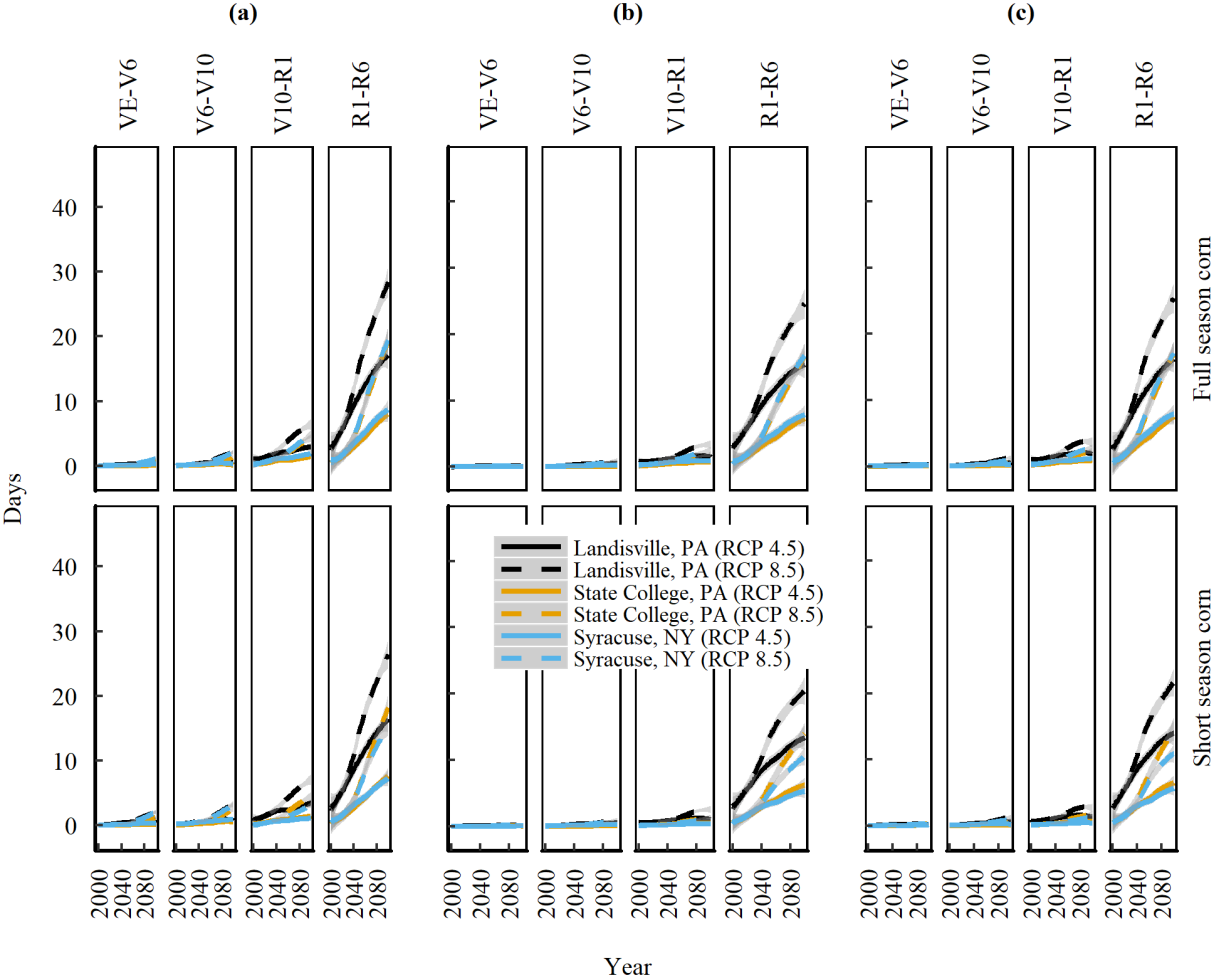
**Trends of projected high temperature frequencies (daily Tmax ≥ 35°C) in the 21**^**st**^ **century during corn growth stages** of emergence (VE), six-leaf (V6), ten-leaf (V10), silking (R1), and physiological maturity (R6), for full and short season corn cultivars planted at (a) Business-as-usual dates. (b) 14-days before the last spring freeze. (c) 1-day after the last spring freeze, at three locations in the Northeast United States under emission scenarios of RCP 4.5 and 8.5. Solid and dashed lines are median trends of data predicted by nine Global Climate Models and shaded areas represent standard error of the mean.

**Fig 5:**
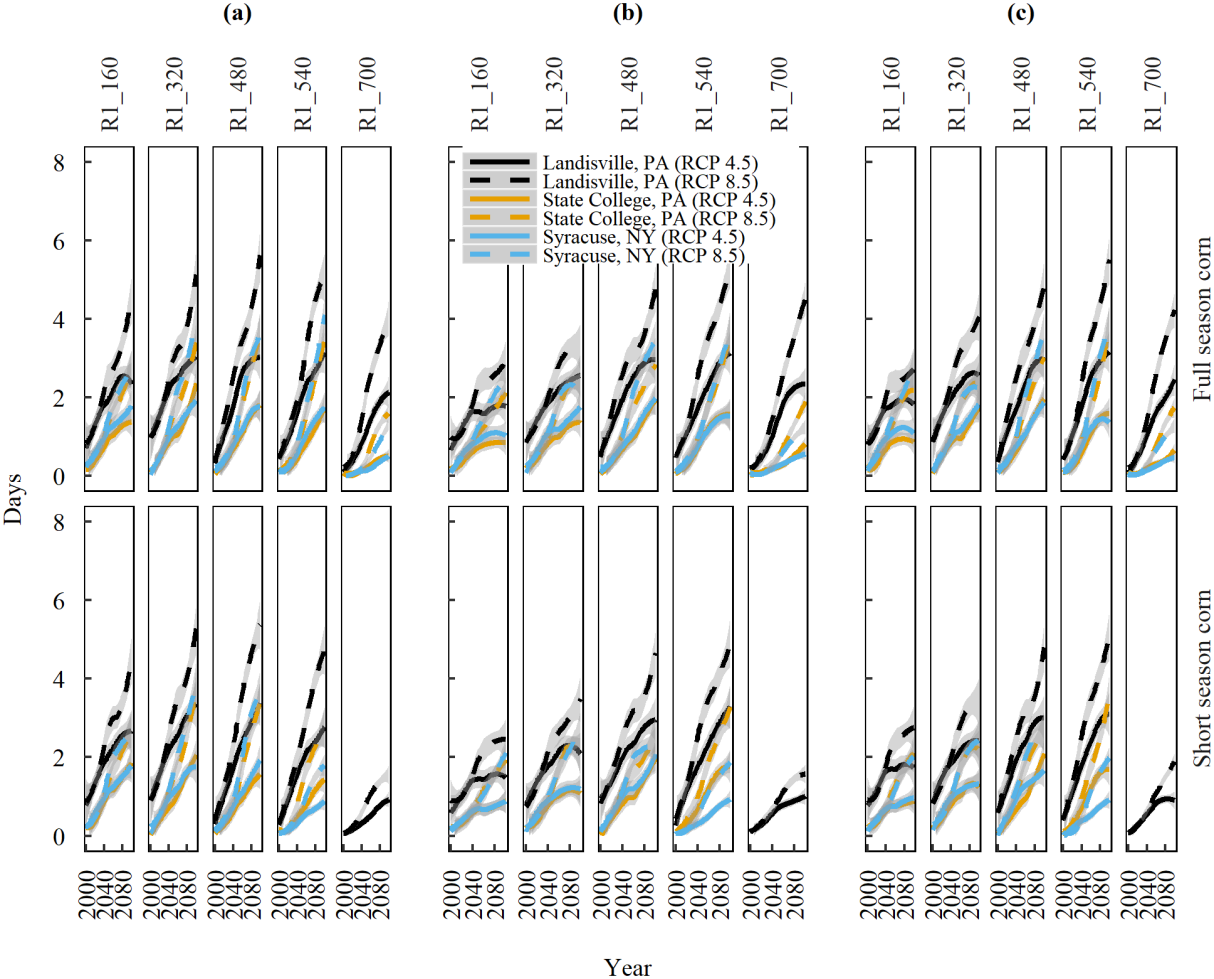
**Trends of projected high temperature frequencies (daily Tmax ≥ 35°C) in the 21**^**st**^ **century during silking (R1) through physiological maturity (R6) at160 GDD intervals for full and short season corn cultivars** planted at (a) Business-as-usual dates. (b)14-days before the last spring freeze. (c)1-day after the last spring freeze, at three locations in the Northeast United States under emission scenarios of RCP 4.5 and 8.5. Solid and dashed lines are median trends of data predicted by Global Climate Models and shaded areas represent standard error of the mean.

Corn planted according to BAUPD in the NE is expected to experience episodes of extreme temperature frequency (daily Tmax > 35°C) (ETF) during several of its growth stages and the ETF varies between locations and RCP (Figs 4 and 5). While the growth stages of VE-V6 and V6-V10 experience minimal ETF (< 2 days), the stages of V10-R1 and R1-R6 suffer from an increasing ETF of 7 days during V10-R1 and 30 days during R1-R6 under RCP 8.5 towards the end of 21st century (Fig 4). When averaged across full and short season corns during V10-R1, the median ETF at Landisville is 1.4 fold greater than State College and 1.6 fold greater than Syracuse under RCP 8.5 (Table 5). The median ETF during R1-R6 is 1.9 fold greater at Landisville compared to State College and 1.8 fold greater than Syracuse under RCP 8.5. When compared between RCPs, the slope is steeper for RCP 8.5 (Fig 4). The median ETF under RCP 8.5 during V10-R1 is 3.4 fold greater at Landisville, 5.7 fold greater at State College and 3.6 fold greater at Syracuse compared to RCP 4.5. During R1-R6, the median ETF under RCP 8.5 is 3.1, 15 and 8 fold greater at Landisville, State College and Syracuse, respectively, compared to RCP 4.5 (Table 5).

**Table 5:** Projected rates of extreme temperature frequencies (daily Tmax ≥ 35°C) (day decade^-1^) during the 21^st^ century in two important growth stages of corn cultivars planted according to the business-as-usual date, scenario-1 (14 days before the last spring freeze), and scenario-2 (1 day after the last spring freeze) at three locations in the Northeast United States, under two emission scenarios. Rates are reported as median values across projected data from 9 Global Climate Models.

			Landisville		State College		Syracuse	
Growth Stage	Planting date	Corn cultivar	RCP 4.5	RCP 8.5	RCP 4.5	RCP 8.5	RCP 4.5	RCP 8.5
V10-R1	Business-as-usual	Full season	0.13	0.49	0	0.37	0.12	0.41
		Short Season	0.20	0.67	0.13	0.42	0.07	0.30
	Average Business-as-usual		0.17	0.58	0.07	0.40	0.10	0.36
	Scenario 1	Full season	0.09	0.23	0.56	0.11	0.12	0.12
		Short Season	0.32	0.01	0.04	0	0	0
	Scenario 2	Full season	0.16	0.23	0.15	0	0.13	0.33
		Short Season	0.18	0.17	0	0.09	0	0
	Average Scenarios 1 & 2		0.19	0.16	0.19	0.05	0.06	0.11
R1-R6	Business-as-usual	Full season	1.1	2.8	0.2	1.6	0.2	2.0
		Short Season	0.8	2.7	0.0	1.5	0.2	1.2
	Average Business-as-usual		0.9	2.8	0.1	1.5	0.2	1.6
	Scenario 1	Full season	1.0	2.5	0.0	1.1	0.3	1.4
		Short Season	0.6	1.9	0.4	1.1	0.3	0.8
	Scenario 2	Full season	1.0	2.6	0.2	1.2	0.3	1.3
		Short Season	1.2	2.1	0.3	1.2	0.3	0.7
	Average Scenarios 1 & 2		1.0	2.3	0.2	1.2	0.3	1.0

A closer look into the subdivisions of R1-R6 stage (Fig 5) suggests that, towards the end of 21st century (2080–2100), corn cultivars planted according to BAUPD are expected to experience 3 to 6 days of extreme temperature (daily Tmax ≥35°C) at 160 GDD intervals under RCP 8.5. The median slope of ETF varies between locations during each 160 GDD subdivision and is greatest at Landisville (0.56 days per 160 GDD interval per decade) followed by Syracuse (0.49 days per 160 GDD interval per decade) and State College (0.38 days per 160 GDD interval per decade) under RCP 8.5.

Shifting planting dates according to scenarios 1 or 2 helps reduce ETF during the V10-R1 and R1-R6 stages, but the effect of planting date on ETF is stronger for V10-R1 than R1-R6 for both the cultivars at all locations (Table 5, Fig 5b and 5c). Averaged across cultivars, changing planting date from BAUPD to Scenarios 1 and 2, helps reduce the ETF during V10-R1 by 72%, 87.5% and 68.7% at Landisville, State College, and Syracuse, respectively under RCP 8.5. ETF during R1-R6 reduces by 16.6%, 16.6% and 37.5% at Landisville, State College, and Syracuse, respectively, upon shifting the planting dates under RCP 8.5.

Freeze can occur early in the corn growing season after planting (spring freeze) or late in the season before maturity (fall freeze). In either cases, freezing damages the corn plant leading to crop failure or yield loss [59]. We explored the frequencies of daily Tmin ≤ 0°C at each growth stage of full and short season corn cultivars planted according to BAUPD, scenario 1 and scenario 2 dates at all three locations under RCP 4.5 and 8.5. We found no significant trend in occurrence of spring or fall freeze within the boundaries of the corn-growing season (planting to maturity) selected in this study (data not shown). Therefore, the potential is small for corn experiencing freeze in any of its growth stages for the rest of the 21st century.

### Water deficit during corn growth stages

**Fig 6:**
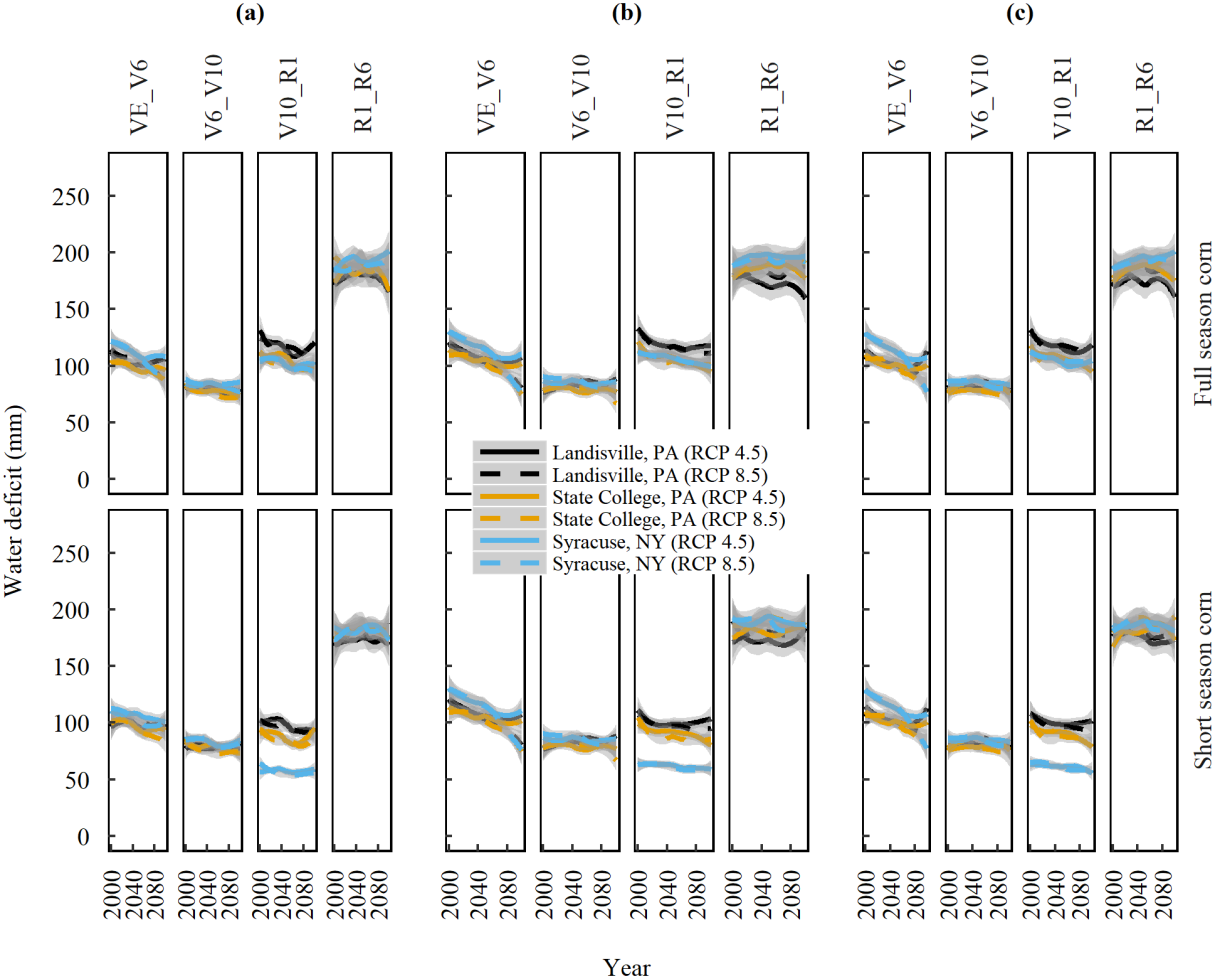
**Trends of projected water deficit (PET-precipitation) in the 21**^**st**^ **century during corn growth stages** of emergence (VE), six-leaf (V6), ten-leaf (V10), silking (R1), and physiological maturity (R6), for full and short season cultivars planted at (a) Business-as-usual dates. b)14-days before the last spring freeze, and (c)1-day after the last spring freeze, at three locations in the Northeast United States under emission scenarios of RCP 4.5 and 8.5. Solid and dashed lines are median trends of data predicted by nine Global Climate Models and shaded areas represent standard error of the mean.

Water deficit (WD), calculated in this study as the difference between PET and precipitation, is an indicator of potential water stress severity. We found a clear distinction in WD between the growth stages for the two emission pathways and planting dates. The WD varied marginally between locations. Averaged among locations and RCPs, the greatest WD was observed at R1-R6 (approximately 200 mm) followed by V10-R1 (approximately 100 mm), VE-V6 (approximately 100 mm), and V6-V10 (approximately 80 mm) (Fig 6).

**Fig 7:**
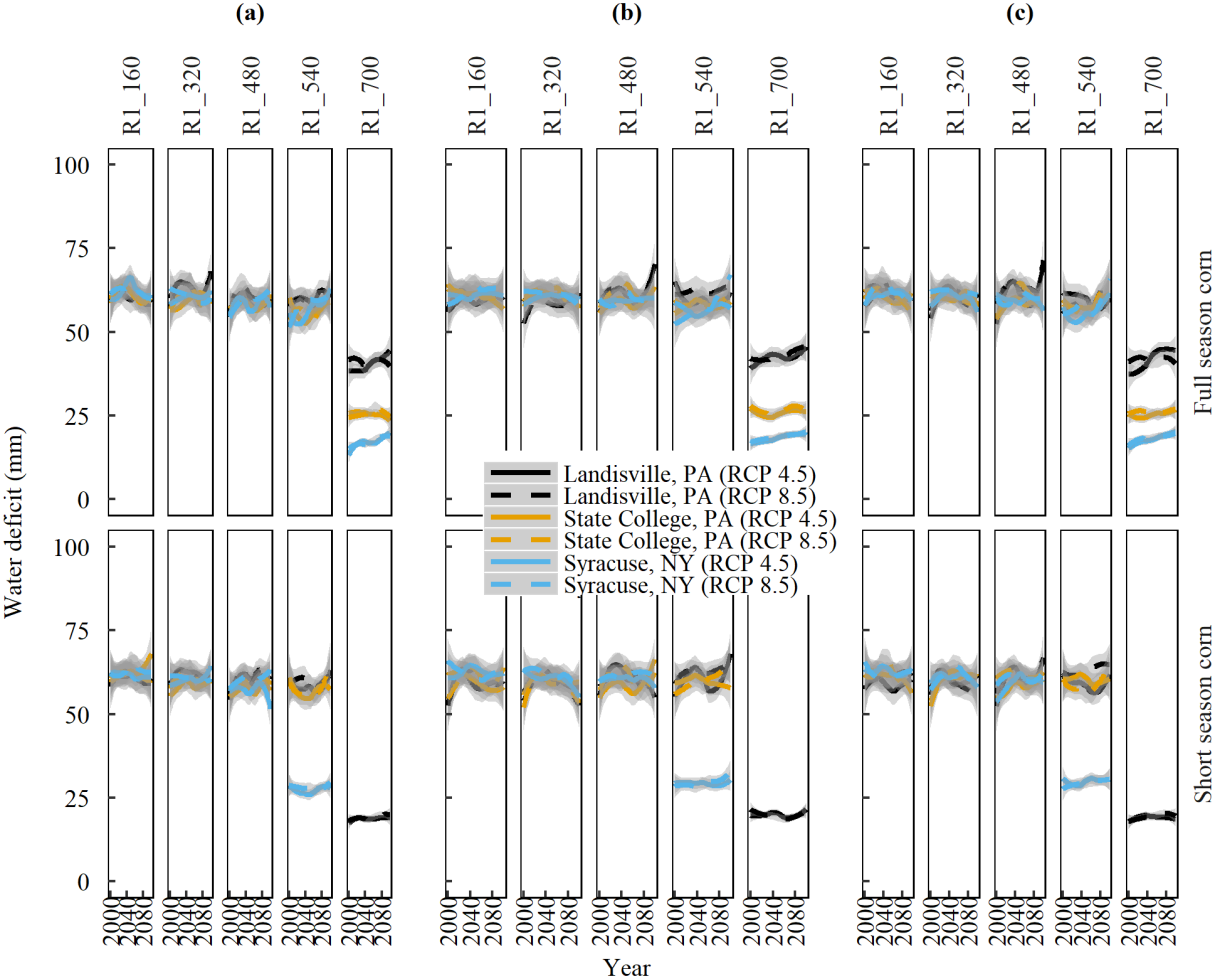
**Trends of projected water deficit (PET-precipitation) in the 21**^**st**^ **century during silking (R1) through physiological maturity (R6) at160 GDD intervals for full and short season corn cultivars** planted at (a) Business-as-usual dates. (b)14-days before the last spring freeze, and (c)1-day after the last spring freeze, at three locations in the Northeast United States under emission scenarios of RCP 4.5 and 8.5. Solid and dashed lines are median trends of data predicted by nine Global Climate Models and shaded areas represent standard error of the mean.

Since there are subtle differences between the two RCP, hereafter, we report the values of RCP 8.5. Our analysis reveals that long-term WD trends within the growth stages are either positive, negative, or not significant between locations (Table 6). The relative rate in the difference between PET and precipitation is what determines the overall direction (sign) and strength of the WD. For example, if PET increases at a greater rate than precipitation, then water deficit exhibits an increasing trend. In contrast, if precipitation increases more rapidly than PET, then WD declines. A non-significant trend indicates no change in WD with time. The WD during R1-R6 stage shows a positive trend for full season corn at State College and Syracuse with the exception at Landisville, which shows no significant trend. Contrary to this, WD during R1-R6 stage shows a negative trend at all three locations. This reversal in sign (direction) of trend indicates that short season corn, irrespective of the location will potentially experience lesser WD during the R1-R6 stages. A closer look into the 160 GDD subdivisions of the R1-R6 stage indicates that the WD averages 55 mm across all locations until the accumulation of 540 GDD heat units and then declines (Fig 7).

**Table 6:** Rate of projected water deficit (mm decade^-1^) in the 21^st^ century during vegetative and reproductive stages of corn cultivars planted according to business-as-usual date, at three locations in the Northeast United States under the emission scenario RCP 8.5. Rates are reported as median values across projected data from 9 Global Climate Models.

		Landisville	State College	Syracuse
Growth Stage	Corn cultivar	RCP 8.5	RCP 8.5	RCP 8.5
VE-V6	Full season	0	0	0
	Short season	-0.4	-0.4	-0.5
V6-V10	Full season	0	-0.4	0
	Short season	-0.4	0	-0.3
V10-R1	Full season	-0.4	0.0	-0.5
	Short season	-0.1	-0.3	-0.2
R1-R6	Full season	0	6.0	8.0
	Short season	-9.0	-8.0	-5.0

Shifting the planting dates according to scenarios 1 or 2 did not offer a significant relief in WD for any of the growth stages or corn cultivars at the three locations. Irrigation would be required to reduce the water deficit, and may prove beneficial to combat heat and water stress during the R1-R6 stages.

## Discussion

Earlier studies on corn have focused on quantification of yield losses due to climate change [1, 15, 34] but overlooked the growth and developmental aspects resulting from extreme climate at local levels. By using downscaled climate projections, this study quantifies the impacts of extreme climate on corn growth and development for the 21st century in the NE US and for the first time characterizes the locational differences in spring and fall freeze recession rate, rate of GDD accumulation and their effect on corn maturity, frequencies of high temperature (ETF) as well as water deficits during different stages of corn growth and development. We demonstrate that corn production in northern locations of the NE US will potentially experience greater changes in heat effects under business-as usual management practices as indicated by the faster GDD accumulation rate and more frequent high temperatures and drought conditions during the silking-anthesis stage than found in the southern parts of the region.

Our analysis indicates that current or business-as usual management practices would require a change, and adaptation strategies would vary between locations. The faster rates of recession of last spring freeze under RCP 8.5 and proximity of recession rates of northern and central locations of the NE to southern counterparts further indicate spatial differences and a greater warming rate in higher latitudes (Syracuse and State College). Earlier studies such as Cooter and Leduc [7] examined the frost date trends in the New England region of the NE US from 1950 to 1990 and found that the last spring freeze occurred 11 days earlier in the mid-1990s than in the 1950s. Wake [8] also noted that the growing season length in the NE US has increased by 8 days due to recession of the last spring freeze and there was spatial variability with some locations experiencing considerably longer growing seasons. Our results agree with the trends found in the previous climate change studies conducted in the NE (e.g. [7, 9]).

Recession of spring freeze dates will allow an extended freeze free growing season and greater opportunity for double cropping towards the end of 21st century. These benefits may be masked though due to a faster GDD accumulation rate which will shorten the time to attain physiological maturity as well as occurrences of high temperature episodes (daily Tmax > 35°C) during important corn growth stages (such as R1-R6). The WD during R1-R6 stages further complicates the situation since the analysis suggests that WD will be greatest during these stages. Extreme heat alone can raise water demand, but can also lower the future water supply by reducing soil water content through raised transpiration rates [15]. Teixeira et al. [60] reported that land areas most affected by heat stress were located in mid to high latitudes in the northern hemisphere (40 and 60°N). Shortening of corn maturity due to heat stress has also been cited by Lobell et al. and Luo [15, 24]. Zhenong et al. [2] reported an average reduction of 25% in corn grain filling days due to heat stress under RCP 8.5 towards the end of 21st century (2080–2100). Faster GDD accumulation forces the plant to complete its lifecycle early, but can also penalize yield by affecting the metabolic processes and reducing carbon assimilation. In a study by Olesen and Bindi [61], a summer heat wave in Europe reduced cereal grain (wheat and corn) production by 23 MT and the yield reduction was attributed to the shorter growing season combined with a higher frequency of maximum temperatures and longer dry spells.

Frequent high temperature exposure during the R1-R6 stage is detrimental for corn plant growth and yield. The interval between anthesis and silking is most sensitive to high temperatures since success of pollination is critical to kernel set, which determines the final yield [19]. The key events for successful kernel set include production of viable pollen, pollen shed and interception by silks, fertilization, and endosperm development [62]. Pollen shed usually lasts up to two weeks during which the silks also elongate until they are pollinated. The period before pollen shed (at least one week), between pollen shed and silk emergence (anthesis-silking interval), and after silk emergence (at least 2 weeks) are critical for kernel set [62]. Concurrent drought and high temperature episodes during any of these events can cause yield losses through several mechanisms. For example, frequent high temperature episodes can reduce pollen production and viability [19]. Water stress can delay silking, which may cause a mismatch between pollen shed and silk emergence. Silks that emerge after pollen shed may not be pollinated resulting in a barren ear. Additionally, high temperature episodes can also reduce silk receptivity, thus preventing pollination or causing successfully pollinated kernels to abort [62]. Temperature stress during the endosperm division phase reduces the potential kernel growth rate and final kernel size [25].

In a US national corn study, Lobell and Asner [34] found a significant correlation between corn yield and observed temperature trends and reported a 17% decrease in corn yield for each 1°C increase in mean growing season temperature. Our analysis indicates that corn production in the NE US, based on current management practices, will potentially experience high temperature episodes and water deficits between stages V10 to R6. Although we did not intend to predict yield, the agronomic anomalies such as ETF and water deficit during key corn stages provide evidence of possible yield losses by mid-century.

Thus, management practices should focus on selecting planting dates that help shift the ETF away from the critical R1 stage to prevent pollen desiccation. If the planting date of scenario 1 is selected in the future, the ETF will shift away from the R1 stage (Fig 5b). Overall, a shift in planting date will be required towards the end of 21st century (2080–2100) to avoid high temperature episodes during the critical stage of pollen formation and fertilization, especially under RCP 8.5. Many corn studies have demonstrated that the decision to plant earlier has provided yield increases [63–64]. For example, Kucharik [65] found that multidecadal trends of earlier planting contributed to rising yields (0.06 and 0.14 Mg ha^-1^ for each additional day of earlier planting) during 1979 to 2005 in 6 out of 12 central US states. Early planting offers several benefits such as an extended growing season allowing corn plants to accumulate more photosynthates, attain physiological maturity before the killing fall frost, and flowering before midsummer heat stress [65–66]. Our study indicates that either of the earlier planting date scenarios could help avoid the high temperature episodes during the R1 stage compared to BAUPD.

While several process-based modelling studies of corn have quantified yield losses due to weather extremes by focusing on heat and water stress, none of the corn models possess explicit routines that represent the effect of heat on pollen viability or flowering success [15, 67]. It is well known that models represent complicated processes and their outputs have uncertainty associated with them. This also applies to the projections from GCMs, which also have uncertainty; hence caution must be maintained when interpreting results from analyses like those presented herein (e.g. [68]). The causes of uncertainty could be many, including the climate forcing data used, downscaling method, etc. In several studies, where processed-based crop models were used in conjunction with the outputs from GCMs, the uncertainty was amplified [69–70]. Additionally, using one specific climate model might lead to contradictory conclusions; hence employing a multi-model ensemble of GCMs should give better representativeness of future projections [36, 70]. Use of multi-model ensemble projections have shown agreement in the direction and magnitude of temperature changes; however, precipitation trends vary [71]. In this study, we used projections from nine climate models, which in itself provides robustness and captures the range of responses of the climate models to RCP scenarios.

Our analysis shows that shifting planting dates in the NE US will help avoid high temperature episodes during the R1-R6 development stages as well as reduce faster developmental rate and maintain normal maturity length. Further, irrigation may be required to reduce the water deficit during the critical stage of anthesis-silking to counteract some of the effects of heat and water stress towards the end of the 21st century. We conclude that a better understanding of climate extremes at local levels is required to determine the right management strategies to adapt to a changing climate.
